# Migration-related determinants of health-care service utilization among persons with a direct migration background in Germany: an exploratory study based on the German Socio-Economic Panel (SOEP)

**DOI:** 10.1007/s10198-024-01708-9

**Published:** 2024-07-15

**Authors:** Thomas Grochtdreis, Hans-Helmut König, Judith Dams

**Affiliations:** Department of Health Economics and Health Services Research, Hamburg Center for Health Economics, University Medical Center Hamburg-Eppendorf, Hamburg, Germany

**Keywords:** Health-care service utilization, Social determinants, Surveys and questionnaires, Transients and migrants, I14, I15

## Abstract

**Background:**

It is known that the health-care service utilization in primary care of persons with a direct migration background is lower compared to non-migrants. However, potential migration-related determinants of health-care service utilization are not known. Therefore, this study aimed to analyze the associations between health-care service utilization and migration-related characteristics of persons with a direct migration background in Germany.

**Methods:**

The migration samples (M1 and M2) of the German Socio-Economic Panel (SOEP) were used as the sample for this study. Associations between the number of visits to primary care physicians in the previous three months and migration-related characteristics were examined using generalized linear models. Associations between the hospitalization within one year and migration-related characteristics were examined using logit models.

**Results:**

The mean number of visits to primary care physicians was about 2, and 8% of persons were hospitalized. Being born in a country other than Russia was associated with a higher number of visits to primary care physicians (+ 26% to + 34%). Both, a very strong connectedness with the country of birth and very good oral German language skills were associated with higher number of visits to primary care physicians (both + 13%) compared to no connectedness and fairly bad oral German language skills.

**Conclusion:**

Only the country of birth, connectedness with the country of birth and oral German language skills may be migration-related determinants of health-care service utilization with regard to the number of visits to primary care physicians by persons with a direct migration background in Germany. With regard to hospitalization, no potential migration-related determinants of health-care service utilization could be identified.

**Supplementary Information:**

The online version contains supplementary material available at 10.1007/s10198-024-01708-9.

## Background

In the year 2023, about 16.1 million persons had their own migration experience and were born without German citizenship [1]. These persons with a so-called direct migration background accounted for around 19% of the total German population [1–3]. Over the last 30 years, the number of persons with a direct migration background in Germany has increased tremendously, not least due to the fall of the Iron Curtain and the enlargement of the European Union [4].

This increase in the number of persons with a direct migration background may make known systemic and personal barriers related to health-care service utilization and migration in Europe more perceptible [5]. Such systemic barriers could be, as described in the literature, the reluctance to pay user fees for health-care services or organizational burdens [6–8]. Personal barriers may include a lack of language skills and knowledge of the health-care system in the host country. Compared to persons without migration background, migrants are generally less likely to utilize specialists in outpatient care and screening services, but a higher utilization of emergency services and a higher number of hospitalizations were associated with a migration background [9–13]. It should be noted, however, that the results of systematic reviews on the differences in health-care service utilization between migrant and non-migrant groups vary widely due to the substantial differences in the host countries in Europe and beyond.

An international systematic review came to the conclusion that persons with a migration background neither excessively utilized primary care physicians nor decided against the utilization of primary care physicians compared to non-migrants [12]. However, a stronger primary care system, such as in Europe or Canada has been associated with a higher health-care service utilization among migrant groups. The three systematic reviews that focused on the health-care service utilization of migrants in Europe found contrasting results with regard to the utilization of primary care physicians as well as specialists in outpatient care, yet with a tendency towards a lower utilization of primary care services by migrant groups [9–11]. These systematic reviews also found higher hospitalization rates, higher numbers of nights spent in hospital and a higher utilization of emergency departments among migrant groups compared to non-migrant groups.

Thus, even for Europe, the results of the systematic reviews show large differences in health-care service utilization between migrant and non-migrant groups, which vary widely due to the diversity of the host countries. For Germany alone, one systematic review found a lower utilization of specialists in outpatient care among persons with migration background compared to non-migrants [13]. No clear evidence was found with regard to utilization of primary care physicians, emergency services and hospitalization. However, persons with a direct migration background, among others, were identified as migrant groups with a particularly low health-care service utilization.

While the studies in the aforementioned systematic reviews concentrated on the comparison of health-care service utilization of persons with migration background and non-migrants and their differences in sociodemographic characteristics, migration-related characteristics are rarely incorporated in the analyses as potential determinants of health-care service utilization. According to the behavioral model of health-care service utilization by Andersen [14–17], migration background, in the sense of the respective country of birth, is an individual predisposing factor for health-care service utilization. However, persons with a direct migration background are very heterogeneous in terms of migration-related characteristics, so that health-care service utilization can be determined by different migration-related predisposing, enabling and need factors. According to Norredam et al. [11], migration-related predisposing factors, especially for persons with a direct migration background, include language skills and cultural barriers. In addition, the time since the migration to the host country and the main reason for migration were also identified as migration-related determinants of health-care service utilization and health outcomes [18–22]. Other determinants related to migration and ethnicity may include the connectedness with the country of birth and the host country as well as disadvantages due to origin in the host country.

With regard to health-related quality of life, potential migration-related determinants have already been identified in persons with a direct migration background in Germany [23]. In addition to the country of birth, the time since migration to Germany and the relationship status before migration were associated with health-related quality of life. To our knowledge, however, potential migration-related determinants of health-care service utilization among persons with a direct migration background have not yet been identified for Germany. Therefore, the aim of this study was to analyze the associations between health-care service utilization and migration-related characteristics of persons with a direct migration background in Germany.

## Methods

### Sample

The German Socio-Economic Panel (SOEP) provided by the German Institute for Economic Research (DIW Berlin) was supplemented with two additional migrant samples in 2013 and 2015 (M1 and M2) [24]. By 2023, seven waves (31 to 37) were available, covering the years 2014 to 2020 (*n* = 7391, 36,119 observations). From the available waves, the adult sample with a direct migration background without missing information on the number of visits to primary care physicians and number of nights spent in hospital was used to generate an analysis sample (*n* = 4206, 15,837 observations; 44% of the original sample). In this context, persons with a direct migration background were defined as persons with their own migration experience born without German citizenship, based on a predefined variable of the SOEP, which combined information on country of birth, citizenship and parents [25].

### Measures

Participants in the SOEP were asked about the number of visits to primary care physicians in the previous three months. No distinction was made between primary care physicians and specialists in outpatient care. Furthermore, participants were asked about the number of nights they had spent in hospital within one year. Based on this, it was determined whether the participants had visited a doctor in the previous three months (yes/no) or they had spent at least one night in hospital within one year (yes/no).

The following self-reported migration-related characteristics were derived from the SOEP: country of birth, time since the migration to Germany, the main reason for migration, connectedness with the country of birth (How connected do you feel to your country of birth? Very strongly to not at all), feeling German (To what extent do you feel German? Entirely to not at all), disadvantages due to origin (How often have you personally experienced being disadvantaged in Germany because of your origin? Often, rarely, never), and oral German language skills. The self-created questions with regard to the migration-related characteristics in the SOEP were based on an approach developed by Esser [26], which emphasizes the multidimensional character of integration with regard to cognitive, structural, social and identificatory dimensions [27]. Thereby, the connectedness with the country of birth and feeling German can be seen as migration-specific key indicators for identificatory integration, alongside cultural and linguistic integration.

The country of birth was categorized according to the frequency of occurrence into Russia, Romania, Kazakhstan, Türkiye, another Eastern European country, another European country, an African country, another Asian country, and an American/Oceanic country according to the United Nations Standard Country or Area Codes for Statistical Use (M49) [28]. The oral German language skills were derived from the question ‘How well can you speak German?’, which were rated on a 5-point Likert scale ranging from 1 (Very good) to 5 (Not at all).

The current self-reported health status of the SOEP participants was derived from the question ‘How would you describe your current health?’, which was rated on a 5-point Likert scale ranging from very good to bad health. It has been shown that self-reported health correlates highly with more objective health measures (e.g., [29, 30]. Furthermore, self-reported sex, age, marital status, school-leaving qualification, employment, nationality, and religious affiliation were derived from the SOEP as sociodemographic characteristics.

### Statistical analysis

In order to improve accuracy and statistical power, multiple imputation by chained equations with predictive mean matching and a total number of m = 20 imputations was used for the 23 relevant variables. Missing information varied between 0.00% and 10.30%, and 5625 of all 364,251 records (1.54%) were incomplete.

Descriptive statistics of migration-related and sociodemographic characteristics of the sample were calculated based on cross-sectional data, whereby data from the first survey after inclusion of the respective person in the SOEP was used. Differences in the proportion of persons with at least one visit to a primary care physicians, the (zero-truncated) mean number of visits to primary care physicians, the proportion of persons with at least one night in hospital, and the (zero-truncated) mean number of nights in hospital were analyzed using F-tests.

Associations between the number of visits to primary care physicians (or the number of nights spent in hospital) and migration-related characteristics, current health status, and sociodemographic characteristics were examined using generalized linear models with gamma family and log link function with cluster robust standard errors for population-averaged panel data. Associations between the hospitalization (yes/no) (the utilization of a doctor (yes/no)) and migration-related characteristics, current health status, and sociodemographic characteristics were examined using random effects panel data logit models with cluster robust standard errors. As covariates, the migration-related characteristics ‘time since the migration to Germany’, ‘German citizenship’, ‘main reason for migration’, ‘connectedness with the country of birth’, ‘feeling German’, ‘disadvantages due to origin’, and the ‘oral German language skills’ were added to the regression models. Furthermore, current health status and the sociodemographic characteristics ‘sex’, ‘age’, ‘employment’, ‘school-leaving qualification’, ‘country of birth’, and ‘religious affiliation’ were included as covariates in all regression models. A test to see if the data met the assumption of collinearity indicated that multicollinearity was probably not a concern (mean VIF = 1.75).

As supplementary analyses, the number of visits to primary care physicians and hospitalization was examined using a generalized linear model and logit model without the COVID-19 pandemic year 2020, respectively. As a further supplementary analysis, the zero-truncated number of visits to primary care physicians (the zero-truncated number of nights spent in hospital) was examined using a generalized linear model with gamma family and log link function with cluster robust standard errors for population-averaged panel-data.

All analyses were performed using Stata/MP 18.0 (StataCorp, College Station, TX, USA). All statistics were two-sided with a significance level of *p* < 0.05.

## Results

### Sample characteristics

About half (51%) of the sample (*n* = 4206) was female, the mean age was 39 years and 57% of all persons were between 25 and 44 years old (Table 1). The absolute majority of the sample was married or in a partnership (61%) and the relative majority had a secondary general school qualification (36%). Of all persons in the sample, 34% had a German citizenship and 65% were employed.

The current health status of the persons in the sample was overall very good or good (64%). About 23% of the persons in the sample had a satisfactory health status and 14% had a poor or bad health status. The mean number of visits to primary care physicians for the whole sample was about 2, and 8% of all persons in the sample were (Table 1). The mean number of visits to primary care physicians and the percentage of hospitalization for different current health statuses, and sociodemographic characteristics and migration-related characteristics are shown in Tables S1 and S2 in the online Supplementary Materials.

**Table 1:** Self-reported sociodemographic characteristics of the sample (years 2015 to 2020; *n* = 4206)

Variables	*N* (%) / Mean (SE)
Sex, female	2126 (50.55)
Age, mean	38.70 (0.19)
18–24	546 (12.98)
25–34	1120 (26.63)
35–44	1279 (30.41)
45–45	787 (18.71)
55–64	356 (8.46)
≥ 65	118 (2.81)
Marital status	
Married/in partnership	2595 (61.21)
Never married/single	969 (22.28)
Widowed	273 (6.49)
Separated/divorced	379 (9.02)
School-leaving qualification^1^	
Secondary general school	1525 (36.27)
Secondary school	1045 (24.85)
Academic secondary school	1287 (30.59)
No school-leaving qualification	148 (3.52)
Employment, employed	2743 (65.22)
Citizenship, German	1409 (33.50)
Religious affiliation	
Christian	2237 (53.19)
Muslim	811 (19.27)
Other faith	189 (4.49)
Non-denominational	969 (23.04)
Current health status	
Very good	839 (19.95)
Good	1843 (43.82)
Satisfactory	956 (22.73)
Poor	438 (10.41)
Bad	130 (3.09)
Health-care service utilization	
Number of visits to primary care physicians in the previous three months, mean	1.93 (0.08)
Hospitalization within one year	321 (7.63)

SE: Standard error

^1^ ‘Other school-leaving qualification’ is not shown

The persons in the sample were mainly born in Russia (12%), Türkiye (12%), Romania (9%), and Kazakhstan (9%), while the rest of the sample was born in other European countries (32%) and Asian countries (21%; Table 2). The mean years since migration to Germany in the sample were 13, and the persons in the sample mainly migrated for family and partnership reasons (49%), followed by economic reasons (32%) and political reasons (12%). Connectedness with their country of birth was very strong or strong (48%) among the persons in the sample. About 31% of the persons in the sample had only some connectedness with their country of birth and about 20% had little or no connectedness. The relative majority of the sample (39%) was predominantly or entirely connected with Germany in the sense of feeling German. About 37% of the persons in the sample felt German only in some respects and about 23% felt hardly or not at all German. The absolute majority of the sample never experienced disadvantages due to their origin (58%), while 33% rarely and 9% often experienced disadvantages. Oral German language skills were good or very good overall (62%), 27% stated their oral German language skills were not bad and 11% stated their oral German language skills were fairly bad or that they were not able to speak German at all.

**Table 2:** Self-reported migration-related characteristics of the sample (years 2015 to 2020; *n* = 4206)

Variables	*N* (%) / Mean (SE)
Years since migration to Germany, mean	12.53 (0.13)
Country of birth	
Russia	491 (11.67)
Romania	372 (8.84)
Kazakhstan	366 (8.70)
Türkiye	502 (11.94)
Other East European country^1^	420 (9.99)
Other European country^2^	928 (22.06)
African country	154 (3.66)
Other Asian country^3^	877 (20.85)
American/Oceanic country	96 (2.28)
Main reason for migration^4^	
Family/partnership reasons	2067 (49.14)
Economic reasons	1351 (32.12)
Political reasons	488 (11.59)
Connectedness with country of birth	
Very strong	786 (18.69)
Strong	1247 (29.64)
In some respects	1314 (31.24)
Hardly	590 (13.80)
Not at all	279 (6.64)
Feeling German	
Entirely	613 (14.58)
Predominantly	1048 (24.91)
In some respects	1559 (37.06)
Hardly	621 (14.77)
Not at all	365 (8.68)
Disadvantages due to origin	
Often	385 (9.16)
Rarely	1374 (32.67)
Never	2447 (58.18)
Oral German language skills	
Very good	1210 (28.76)
Good	1384 (32.90)
Not bad	1145 (27.23)
Fairly bad	411 (9.78)
Not at all	56 (1.33)

SE: Standard error

^1^ Without Russia, Türkiye, and Romania

^2^ Without East Europe

^3^ Without Kazakhstan

^4^ ‘Other main reason for migration’ is not shown

### Number of visits to primary care physicians

The generalized linear model for the number of visits to primary care physicians showed no associations with the migration-related characteristics ‘time since migration to Germany’, ‘main reason for migration’, ‘feeling German’, and ‘disadvantages due to own origin’ (Table 3). Being born in Romania, Türkiye, other (Eastern) European, Asian countries and Africa was statistically significantly associated with a higher number of visits to primary care physicians compared with being born in Russia (+ 21% to + 34%; all with *p* < 0.050). A weaker connectedness with one’s country of birth was statistically significantly associated with a lower number of visits to (− 9% to − 17%) compared to a very strong connectedness with one’s country of birth (all with *p* < 0.050). Furthermore, poorer oral German language skills were statistically significantly associated with a lower number of visits to primary care physicians, by − 7% to − 13% compared to very good oral German language skills (all with *p* < 0.050).

No associations with the sociodemographic characteristics ‘sex’, ‘age’, ‘marital status’, ‘school-leaving qualification’, ‘citizenship’, and ‘religious affiliation’ were shown in the generalized linear model of the number of visits to primary care physicians. Being employed was statistically significantly associated with a 31% lower number of visits to primary care physicians compared with not being employed (*p* < 0.001). A poorer current health status was statistically significantly associated with a higher number of visits to primary care physicians. For example, a less good current health was associated with a 3.80 times higher number of visits to primary care physicians compared with a very good current health (*p* < 0.001).

The results of the logit model of utilization of a doctor and migration-related characteristics, current health status, and sociodemographic characteristics are shown in Table S3 in the online Supplementary Materials.

### Hospitalization

The logit model of hospitalization showed no associations with any migration-related characteristic except for ‘country of birth’, ‘main reason for migration’, and ‘connectedness with country of birth’ (Table 3). Being born in Türkiye was statistically significantly associated with a higher odds of hospitalization compared with being born in Russia (OR: 1.51, *p* = 0.027). Political reasons as main reason for migration was associated with a lower odds of hospitalization compared with family/partnership as main reason for migration (OR 0.74, *p* = 0.038). Being strongly and in some respects connected with one’s country of birth was statistically significantly associated with a higher odds of hospitalization compared with being very strongly connected with one’s country of birth (OR 1.28 to 1.39, *p* < 0.050).

No associations with the sociodemographic characteristics ‘sex’, ‘age’, ‘school-leaving qualification’, ‘citizenship’, and ‘religious affiliation’ were shown in the logit model of hospitalization. Being never married/single and being widowed was statistically significantly associated with a lower odds of hospitalization compared with being married or in partnership (OR 0.67 and 0.72, both with *p* < 0.050). Being employed was statistically significantly associated with a lower odds of hospitalization compared with not being employed (OR: 0.33, *p* < 0.001). A poorer current health status was statistically significantly associated with a higher odds of hospitalization (OR: 2.28 to 7.79; all with *p* < 0.001). The results of the generalized linear model of the number of nights spent in hospital and migration-related characteristics, current health status, and sociodemographic characteristics are shown in Table S3 in the online Supplementary Materials.

### Supplementary analyses

The COVID-19 pandemic year 2020 was statistically significantly associated with a lower number of visits to primary care physicians (− 24%; *p* < 0.001), yet not with a lower odds of hospitalization compared with the year 2014 and 2015, respectively (Table 3). Analyses without the year 2020 did not alter the results of the generalized linear model of number of visits to primary care physicians and the logit model of hospitalization (data not shown).

The supplementary analyses with zero-truncated number of visits to primary care physicians and zero-truncated number of nights spent in hospital did not alter the results of the generalized linear models (Table S4 in the online Supplementary Materials).

**Table 3:** Model of self-reported number of visits to primary care physicians in the previous three months (*n* = 4206; 15,837 observations), and self-reported hospitalization within one year (*n* = 3711; 13,472 observations) and migration-related characteristics, current health, and sociodemographic characteristics

Variable	Model 1^†^ (dependent variable number of visits to primary care physicians in the previous three months)			Model 2^‡^ (dependent variable hospitalization within one year)		
	Exp(b)	95% CI	*p*-value	OR	95% CI	*p*-value
Current health status (Ref. very good)						
Good	*1.41*	*1.31; 1.52*	*< 0.001*	*1.21*	*0.94; 1.55*	*0.145*
Satisfactory	*2.22*	*2.05; 2.42*	*< 0.001*	*2.28*	*1.72; 3.03*	*< 0.001*
Poor	*3.80*	*3.46; 4.18*	*< 0.001*	*4.72*	*3.34; 6.69*	*< 0.001*
Bad	*6.63*	*5.61; 7.84*	*< 0.001*	*7.79*	*5.28; 11.49*	*< 0.001*
Sex (Ref. male)						
Female	1.00	0.96; 1.05	0.924	0.91	0.78; 1.06	0.229
Age, years	1.00	1.00; 1.00	0.731	1.00	1.00; 1.01	0.285
Marital status (Ref. married/in partnership)						
Never married/single	0.98	0.92; 1.03	0.401	*0.67*	*0.54; 0.83*	*< 0.001*
Widowed	1.09	0.97; 1.21	0.140	*0.72*	*0.52; 0.99*	*0.040*
Separated/divorced	1.03	0.94; 1.14	0.507	0.82	0.62; 1.10	0.191
School-leaving qualification (Ref. secondary general school)^1^						
Secondary school	1.02	0.96; 1.09	0.625	1.07	0.86; 1.33	0.563
Academic secondary school	0.98	0.92; 1.04	0.527	0.97	0.79; 1.19	0.789
No school-leaving qualification	0.94	0.80; 1.11	0.454	0.96	0.53; 1.75	0.896
Employment (Ref. unemployed)						
Employed	*0.69*	*0.66; 0.73*	*< 0.001*	*0.33*	*0.27; 0.39*	*< 0.001*
German citizenship (Ref. yes)						
No	0.99	0.93; 1.06	0.836	1.09	0.87; 1.37	0.439
Religious affiliation (Ref. Christian)						
Muslim	1.04	0.94; 1.15	0.436	0.79	0.60; 1.05	0.102
Other faith	1.02	0.87; 1.18	0.837	0.73	0.47; 1.15	0.176
Non-denominational	1.01	0.94; 1.09	0.750	1.15	0.91; 1.45	0.239
Country of birth (Ref. Russia)						
Romania	*1.26*	*1.11; 1.43*	*< 0.001*	1.09	0.76; 1.57	0.635
Kazakhstan	1.10	0.97; 1.25	0.123	1.08	0.74; 1.56	0.705
Türkiye	*1.27*	*1.14; 1.42*	*< 0.001*	*1.51*	*1.05; 2.18*	*0.027*
Other East Europe^2^	*1.28*	*1.14; 1.44*	*< 0.001*	1.09	0.74; 1.62	0.664
Other Europe^3^	*1.34*	*1.21; 1.49*	*< 0.001*	1.19	0.85; 1.67	0.314
Other Asia^4^	*1.30*	*1.16; 1.46*	*< 0.001*	1.35	0.90; 2.02	0.149
Africa	*1.21*	*1.02; 1.44*	*0.025*	1.41	0.87; 2.30	0.161
America/Oceania	1.22	1.00; 1.50	0.054	0.75	0.37; 1.48	0.402
Time since migration to Germany, years	1.00	0.99; 1.00	0.491	1.00	0.99; 1.01	0.609
Main reason for migration (Ref. family/partnership)^5^						
Economic reasons	0.96	0.89; 1.03	0.216	1.15	0.94; 1.40	0.187
Political reasons	0.96	0.86; 1.07	0.484	*0.74*	*0.56; 0.98*	*0.038*
Connectedness with country of birth (Ref. very strong)						
Strong	*0.87*	*0.81; 0.94*	*< 0.001*	*1.29*	*1.01; 1.65*	*0.041*
In some respects	*0.91*	*0.84; 0.98*	*0.019*	*1.38*	*1.08; 1.76*	*0.011*
Hardly	*0.83*	*0.75; 0.91*	*< 0.001*	1.09	0.80; 1.48	0.592
Not at all	*0.87*	*0.76; 0.99*	*0.042*	0.90	0.64; 1.27	0.561
Feeling German (Ref. entirely)						
Predominantly	0.99	0.92; 1.07	0.774	0.98	0.75; 1.28	0.854
In some respects	1.02	0.94; 0.10	0.621	0.91	0.68; 1.22	0.547
Hardly	1.02	0.92; 1.14	0.648	0.74	0.52; 1.06	0.096
Not at all	1.02	0.91; 1.14	0.717	0.84	0.58; 1.22	0.355
Disadvantages due to origin (Ref. often)						
Rarely	1.04	0.94; 1.15	0.454	0.87	0.60; 1.27	0.461
Never	0.98	0.89; 1.08	0.651	0.78	0.54; 1.12	0.172
Oral ability in the German language (Ref. very good)						
Good	*0.93*	*0.87; 0.99*	*0.032*	1.14	0.90; 1.43	0.283
Not bad	*0.89*	*0.82; 0.96*	*0.002*	1.00	0.77; 1.30	0.987
Fairly bad	*0.87*	*0.78; 0.98*	*0.023*	1.02	0.71; 1.45	0.925
Not at all	0.87	0.55; 1.37	0.552	1.03	0.53; 2.02	0.922
Survey year (Ref. 2014/2015^6^)						
2015	1.00	0.93; 1.07	0.934	-	-	-
2016	1.01	0.93; 1.09	0.857	1.00	0.91; 1.31	0.997
2017	0.98	0.91; 1.05	0.554	0.81	0.84; 1.23	0.085
2018	0.98	0.91; 1.06	0.566	0.82	0.75; 1.13	0.206
2019	0.99	0.92; 1.08	0.889	*0.72*	*0.78; 1.20*	*0.021*
2020	*0.84*	*0.77; 0.93*	*< 0.001*	0.76	0.62; 0.99	0.064
Constant	1.13	0.93; 1.39	0.224	0.79	0.40; 0.55	0.490

OR: odds ratio; CI: confidence interval

^1^ ‘Other school-leaving qualification’ is not shown

^2^ Without Russia, Türkiye, and Romania

^3^ Without East Europe

^4^ Without Kazakhstan

^5^ ‘Other main reason for migration’ is not shown

^6^ As no data on the hospitalization was available for the year 2014, observations from 2014 were not used in model 2

^†^ Generalized linear panel-data model with gamma family and log-link function and cluster-robust standard errors for population-averaged panel data

^‡^ Random-effects panel-data logit model with cluster-robust standard errors

## Discussion

The aim of this study was to analyze the associations between health-care service utilization and migration-related characteristics of persons with a direct migration background in Germany. The country of birth was relevant for the frequency of utilization of primary care physicians. In this regard, Russian migrants had a lower utilization of primary care physicians than Romanian and Turkish migrants, and migrants from other (Eastern) European and Asian countries.

The lower utilization of primary care physicians among Russian migrants contrasts with a lower physical health-related quality of life compared to other migrants that was shown in the literature [23]. Thus, despite having a lower physical health-related quality of life, health status and life satisfaction, Russian migrants are less likely to utilize primary care physicians. Conclusively, there might be an actual under-utilization of health-care services among Russian migrants [31]. However, a lower health status among Russian migrants might also result from a lower utilization of primary care physicians. The participants in the SOEP have neither been asked about the spatial characteristics of the area in which they lived before migration to Germany, e.g. rurality or density of health-care services, nor were data on regional allocation of the place of residence of the participants in the SOEP available. Thus, no statements can be made about the heterogeneity of the association between the country of birth and health-care service utilization, even if this is quite likely. Likewise, no statements can be made about the heterogeneity of the effects by the area of destination, such as rurality or density of hospitals and primary care physicians in the place of residence of the participants.

The connectedness with the country of birth could be seen as a cultural barrier or an indicator of acculturation, and thus as a migration-related predisposing factor for health-care service utilization [11, 19]. Interestingly, in the current study, persons with a direct migration background who were very strongly connected with their country of birth utilized primary care physicians more often than persons with a weaker connectedness with their country of birth. In contrast, the preventive utilization of primary care services among migrant workers in Belgium who migrated to Europe in the second half of the 20th century was negatively associated with increasing acculturation [19]. This comparison could give an indication of the differences in health-care service utilization between the migrant groups then and in the last 30 years.

In the current study, the time since the migration to Germany and the main reason for migration were not identified as potential migration-related determinants of health-care service utilization, as no associations were found with either utilization of primary care physicians or hospitalization [18–20, 22]. However, a study analyzing the determinants of mental health-care service utilization among Chinese migrants in Canada found a positive association between time since the migration to Canada and the utilization of mental health specialists in outpatient care [16, 21].

Oral German language skills being a potential determinant of health-care service utilization, as hypothesized in a systematic review [11], was confirmed in the current study, with poorer language skills being associated with a lower utilization of primary care physicians. Thereby, language skills can be seen as an informal facilitator of access to health-care [32, 33]. However, the Canadian study that focused on Chinese migrants found no association between language skills and utilization of mental health-care services [16, 21]. Again, no statements can be made about the likely heterogeneity of the association between the oral German language skills and health-care service utilization. Thereby, the true acting mechanisms in the under-utilization, e.g. with regard to the effect of the socio-demographic characteristics of the person with poorer language skills, stay unknown.

Encouragingly, in the current study, disadvantages due to one’s own origin were not associated with a lower health-care service utilization, suggesting that, contrary to previous assumptions, experiences of racial discrimination may neither be directly related to the health-care system nor indirectly lead to avoidance behavior of health-care services [13, 34].

With regard to non-migration-related predisposing factors for health-care service utilization, no associations were found between utilization of primary care physicians or hospitalization and sex, age, school-leaving qualification, citizenship, and religious affiliation. However, employment was associated with both a lower utilization of primary care physicians and a lower odds of hospitalization. Furthermore, being never married, single or widowed was associated with a lower odds of hospitalization. A systematic review that identified frequently researched predisposing, enabling and need factors for health-care service utilization according to Andersen’s behavioral model found that sex, age, marital status and school-leaving qualification were repeatedly associated with health-care service utilization [16]. In the current study, the inclusion of migration-related characteristics in the models of health-care service utilization meant that certain socio-demographic characteristics such as age and citizenship, but not school-leaving qualifications, were no longer associated with health-care service utilization. This indicates that a part of the variance of the health-care service utilization may be explained by migration-related characteristics rather than by socio-demographic characteristics among persons with a direct migration background in Germany.

### Generalizability

Generalizability of the results of the present study with regard to associations between health-care service utilization and migration-related characteristics to persons with a direct migration background in Germany is basically given, as the data of the SOEP is claimed to be representative of German households. However, in the migration samples of the SOEP, certain persons with a direct migration background, such as those from the new Eastern EU countries and the southern EU member states, were deliberately oversampled [24]. Furthermore, it is possible that certain groups of persons with a direct migration background, such as those who are difficult to reach for sampling to the panel due to a lack of a centralized register of persons with a migration background in Germany or due missing address information, e.g. due to homelessness or lack of legal residence status, were not included in the migration sample [24]. For the sampling of the migrant population for the SOEP, however, it was possible to use a database that covers employees, unemployed persons, job seekers, recipients of unemployment benefits and participants in active labor market programs [24].

### Policy implications

The likely under-utilization of primary care physicians by persons with a direct migration background with poor oral German language skills should be addressed by providing information about the health-care system in Germany in the languages of common countries of origin [5]. In addition to reducing language barriers, the provision of low-threshold health-care services and target group-specific health communication could also be an opportunity [5, 13, 35, 36]. Furthermore, health-care workers should be given access to free translation and professional interpreting services and cultural stereotyping in the health-care system should be minimized [7]. A special focus with respect to access to health-care should be given to persons born in Russia who showed a lower utilization of primary care physicians and a lower physical health-related quality of life as well as to persons born in Türkiye who showed a higher odds of hospitalization [23].

### Strengths and limitations

One major strength of this study was the possibility of presenting the heterogeneity of the group of persons with a direct migration background in terms of migration-related characteristics. This was made possible by the two additional migrant samples that were supplemented to the SOEP [24]. This in turn ensured that persons with migration background were represented proportionally to the German population. Secondly, the SOEP questionnaires were made available in several languages and an interpreter was provided optionally in order to reduce selection bias in favor of persons with better German language skills or a higher level of education.

As a major limitation, it should be mentioned that despite the longitudinal nature of the data, no conclusions could be drawn about the causality of the migration-related determinants of health-care service utilization, as these were largely constant over time and were not considered as fixed effects in the models, and therefore only associations could be analyzed. Furthermore, the current study must be regarded as exploratory rather than confirmatory.

Secondly, the selection of migration-related characteristics, in particular the connectedness with the country of birth, current health status, and sociodemographic characteristics as independent variables in the models of number of visits to primary care physicians and hospitalization might have caused multicollinearity and there is a risk of endogeneity. Furthermore, the use of a large number of independent variables in the models might have introduced overadjustment and increased risk of bias [37]. However, given the exploratory character of the study and the study aim of analyzing associations between health-care service utilization and migration-related characteristics rather than to find causal relationships, potential overadjustment is not considered very serious with regard to false statistical associations.

Thirdly, as already mentioned, the associations of the country of birth, the oral German language skills and also the connectedness with the country of birth with health-care service utilization may probably be heterogeneous with regard to unobserved but also observed socio-demographic characteristics as well as themselves. With regard to observed socio-demographic characteristics, however, interactions with the independent variables ‘country of birth’, ‘connectedness with the country of birth’ and ‘oral German language skills’ were added to the models. Thereby, the sociodemographic characteristics ‘current health status’ and ‘employment’ were selected, as their effects on number of visits to primary care physicians and hospitalization within one year were statistically significant and meaningful (data not shown). Yet, those interaction effects were neither statistically significant nor meaningful.

Fourthly, the associations with health-care service utilization may probably be heterogeneous with regard to unobserved characteristics of health-care services. As no data on regional allocation of the place of residence of the participants in the SOEP was available for the current analyses, it was not possible to add interactions with relevant migration-related characteristics.

Fifthly, no data on hospitalization was available in the SOEP for 2014, meaning that statements on the associations between migration-related characteristics and hospitalization could only be made for the years 2015 to 2020.

Sixthly, 2020 was the first year of the COVID-19 pandemic, and associations between migration-related characteristics and health-care service utilization may have been attenuated in that year. However, repeating the analyses without the year 2020 did not change the associations found between migration-related characteristics and health-care service utilization.

## Conclusion

This study was able to show that the country of birth and the connectedness with the country of birth may be migration-related determinants of health-care service utilization with regard to the number of visits to primary care physicians of persons with a direct migration background. However, with regard to hospitalization, no potential migration-related determinants of health-care service utilization could be identified, except for the fact that being born in Türkiye was positively associated hospitalization compared to being born in Russia. Persons from Russia and Türkiye in particular should be in the focus of health policy with regard to potential underutilization of primary care physicians and over-hospitalization, respectively. Furthermore, the connectedness with the country of birth should be shed in light as a possible cultural barrier or facilitator for health-care service utilization. Further analyses are needed to better understand the causal relationship between potential migration-related determinants and health-care service utilization, and to consider the heterogeneity particularly of associations between the country of birth, the oral German language skills and health-care service utilization in order to be able to better understand the likely underutilization of primary care physicians of persons from Russia, for example. In addition, further analyses should include data on regional allocation of the place of residence of the persons with a direct migration background in order to identify possible interactions of health-care-seeking behavior and the characteristics of health-care services.

## Electronic supplementary material

Below is the link to the electronic supplementary material.

Supplementary Material 1
