# Supplementary material for: Migration-related determinants of health-care service utilization among persons with a direct migration background in Germany: an exploratory study based on the German Socio-Economic Panel (SOEP)
Source: Eur J Health Econ. 2024 Jul 15;26(2):313–23. doi: 10.1007/s10198-024-01708-9 (PMC11889003; doi:10.1007/s10198-024-01708-9)
Supplement: Supplementary file 1 — Supplementary Material 1 [file 10198_2024_1708_MOESM1_ESM.docx]

Table S1: Self-reported visits to primary care physicians within three months and self-reported nights in hospital within one year by sociodemographic characteristics (years 2015 to 2020; n = 4206)

| **Variables** | **N** | **Visits to primary care physicians within three months** | | **Nights in hospital within one year** | |
| --- | --- | --- | --- | --- | --- |
|  |  | Number of persons with at least one doctoral visit (%) | Mean (SE) | Number of persons with at least one night in hospital (%) | Mean (SE) |
| Total sample | 4206 | 2695 (64.08) | 1.93 (0.05) | 321 (7.63) | 0.63 (0.08) |
| Current health |  |  |  |  |  |
| Very good | 839 | 419 (49.94)*** | 1.00 (0.10)*** | 54 (6.44)*** | 0.38 (0.17)*** |
| Good | 1843 | 1087 (58.98) | 1.31 (0.07) | 108 (5.86) | 0.33 (0.11) |
| Satisfactory | 956 | 683 (71.44) | 2.15 (0.10) | 71 (7.43) | 0.50 (0.16) |
| Poor | 438 | 383 (87.44) | 4.08 (0.14) | 62 (14.16) | 1.58 (0.23) |
| Bad | 130 | 123 (94.62) | 7.69 (0.26) | 26 (20.00) | 4.07 (0.43) |
| Sex |  |  |  |  |  |
| Female | 2126 | 1376 (64.72) | 1.98 (0.07) | 167 (7.86) | 0.64 (0.11) |
| Male | 2080 | 1319 (63.41) | 1.87 (0.07) | 154 (7.40) | 0.61 (0.11) |
| Age |  |  |  |  |  |
| 18-24 | 546 | 319 (58.42)*** | 1.58 (0.14)*** | 42 (7.69) | 0.52 (0.21) |
| 25-34 | 1120 | 673 (60.09) | 1.66 (0.10) | 103 (9.20) | 0.50 (0.15) |
| 35-44 | 1279 | 808 (63.17) | 1.86 (0.09) | 96 (7.51) | 0.65 (0.14) |
| 45-45 | 787 | 532 (67.60) | 2.25 (0.12) | 45 (5.72) | 0.70 (0.18) |
| 55-64 | 356 | 270 (75.84) | 2.58 (0.17) | 24 (6.74) | 0.93 (0.26) |
| ≥ 65 | 118 | 93 (78.81) | 2.57 (0.30) | 11 (9.32) | 0.63 (0.45) |
| Marital status |  |  |  |  |  |
| Never married/single | 2595 | 1672 (64.45) | 1.88 (0.07) | 197 (7.58) | 0.52 (0.16) |
| Married/in partnership | 969 | 618 (63.77) | 1.91 (0.11) | 66 (6.80) | 0.68 (0.10) |
| Widowed | 273 | 180 (65.80) | 2.32 (0.22) | 19 (6.85) | 0.40 (0.31) |
| Separated/divorced | 379 | 251 (66.25) | 1.84 (0.18) | 41 (10.70) | 0.65 (0.26) |
| School-leaving qualification^a^ |  |  |  |  |  |
| Secondary general school | 1525 | 976 (63.97) | 1.90 (0.09) | 113 (7.40) | 0.57 (0.13) |
| Secondary school | 1045 | 678 (64.91) | 2.00 (0.09) | 83 (7.97) | 0.50 (0.16) |
| Academic secondary school | 1287 | 824 (64.05) | 1.95 (0.24) | 100 (7.78) | 0.77 (0.14) |
| No school-leaving qualification | 148 | 97 (65.45) | 1.98 (0.28) | 10 (6.92) | 0.78 (0.41) |
| Employment |  |  |  |  |  |
| Employed | 2743 | 1654 (60.30)*** | 1.51 (0.06)*** | 141 (5.14)*** | 0.27 (0.09)*** |
| Unemployed | 1463 | 1041 (71.16) | 2.70 (0.08) | 180 (12.30) | 1.29 (0.13) |
| Citizenship |  |  |  |  |  |
| German | 1409 | 908 (64.44) | 1.86 (0.09) | 74 (5.25)*** | 0.37 (0.13)* |
| Other citizenship | 2797 | 1787 (63.89) | 1.96 (0.06) | 247 (8.83) | 0.75 (0.09) |
| Religious affiliation |  |  |  |  |  |
| Christian | 2237 | 1414 (63.20) | 1.87 (0.07)* | 173 (7.75) | 0.59 (0.11) |
| Muslim | 811 | 543 (66.91) | 2.24 (0.12) | 60 (7.34) | 0.60 (0.18) |
| Other faith | 189 | 121 (63.94) | 2.12 (0.24) | 7 (3.76) | 0.18 (0.36) |
| Non-denominational | 969 | 618 (63.74) | 1.76 (0.11) | 81 (8.35) | 0.81 (0.16) |

Comparisons of probabilities of at least one doctoral visit within three months and probabilities of at least one night in hospital within one year by sociodemographic characteristics were analyzed using F tests. Comparison of mean number of doctoral visits within three months and mean number of nights in hospital within one year by sociodemographic characteristics were analyzed using F tests.

SE: standard error

^a^ ‘Other school-leaving qualification’ is not shown

* p<0.05, ** p≤0.01, *** p≤0.001

Table S2: Self-reported visits to primary care physicians within three months and self-reported nights in hospital within one year by migration-related characteristics (years 2015 to 2020; n = 4206)

| **Variables** | **N** | **Visits to primary care physicians within three months** | | **Nights in hospital within one year** | |
| --- | --- | --- | --- | --- | --- |
|  |  | Number of persons with at least one doctoral visit (%) | Mean (SE) | Number of persons with at least one night in hospital (%) | Mean (SE) |
| Years since migration to Germany |  |  |  |  |  |
| 0-4 | 723 | 438 (60.58)** | 1.69 (0.12)*** | 80 (11.07)*** | 0.81 (0.18)* |
| 5-9 | 1069 | 683 (63.89) | 1.87 (0.10) | 108 (10.10) | 0.69 (0.15) |
| 10-14 | 845 | 556 (65.80) | 1.79 (0.11) | 36 (4.26) | 0.27 (0.17) |
| 15-19 | 947 | 583 (61.56) | 1.83 (0.11) | 46 (4.86) | 0.47 (0.16) |
| ≥ 20 | 622 | 435 (69.94) | 2.62 (0.13) | 51 (8.20) | 1.03 (0.20) |
| Country of birth |  |  |  |  |  |
| Russia | 491 | 305 (62.12)* | 1.66 (0.15)*** | 27 (5.50)* | 0.38 (0.22) |
| Romania | 372 | 223 (59.95) | 1.52 (0.17) | 37 (9.95) | 0.49 (0.26) |
| Kazakhstan | 366 | 228 (62.30) | 1.67 (0.17) | 19 (5.19) | 0.37 (0.26) |
| Türkiye | 502 | 313 (62.35) | 1.96 (0.15) | 51 (10.16) | 1.14 (0.22) |
| Other East European country^a^ | 420 | 265 (63.10) | 1.78 (0.16) | 40 (9.52) | 0.57 (0.24) |
| Other European country^b^ | 928 | 601 (64.76) | 2.03 (0.11) | 66 (7.11) | 0.63 (0.16) |
| African country | 877 | 105 (68.18) | 2.51 (0.26) | 14 (9.09) | 0.68 (0.40) |
| Other Asian country^c^ | 154 | 601 (68.53) | 2.26 (0.11) | 63 (7.18) | 0.70 (0.17) |
| American/Oceanic country | 96 | 54 (56.25) | 1.32 (0.33) | 4 (4.17) | 0.15 (0.50) |
| Main reason for migration^d^ |  |  |  |  |  |
| Family/partnership reasons | 2067 | 1327 (64.20)* | 1.92 (0.07) | 157 (7.61)* | 0.60 (0.11) |
| Economic reasons | 1351 | 831 (61.52) | 1.80 (0.09) | 123 (9.07) | 0.79 (0.14) |
| Political reasons | 488 | 330 (67.71) | 2.26 (0.15) | 29 (6.03) | 0.51 (0.23) |
| Connectedness with country of birth |  |  |  |  |  |
| Very strong | 786 | 533 (67.77) | 2.25 (0.12)* | 64 (8.14) | 0.59 (0.18) |
| Strong | 1247 | 794 (63.66) | 1.71 (0.09) | 99 (7.96) | 0.51 (0.14) |
| In some respects | 1314 | 846 (64.42) | 1.97 (0.09) | 96 (7.34) | 0.70 (0.14) |
| Hardly | 590 | 364 (61.69) | 1.88 (0.14) | 41 (6.95) | 0.74 (0.21) |
| Not at all | 279 | 164 (58.88) | 1.86 (0.20) | 21 (7.54) | 0.65 (0.30) |
| Feeling German |  |  |  |  |  |
| Entirely | 613 | 375 (61.20) | 1.86 (0.13)* | 47 (7.67) | 0.56 (0.20) |
| Predominantly | 1048 | 662 (63.15) | 1.75 (0.10) | 70 (6.71) | 0.58 (0.15) |
| In some respects | 1559 | 994 (63.79) | 1.91 (0.08) | 116 (7.42) | 0.72 (0.13) |
| Hardly | 621 | 415 (66.83) | 2.04 (0.13) | 54 (8.77) | 0.58 (0.20) |
| Not at all | 365 | 248 (68.08) | 2.38 (0.17) | 33 (9.16) | 0.53 (0.26) |
| Disadvantages due to origin |  |  |  |  |  |
| Often | 385 | 251 (65.19) | 2.31 (0.17)* | 26 (6.74) | 1.14 (0.26) |
| Rarely | 1374 | 893 (65.02) | 2.00 (0.09) | 99 (7.72) | 0.55 (0.14) |
| Never | 2447 | 1551 (63.37) | 1.83 (0.07) | 189 (7.73) | 0.58 (0.10) |
| Oral ability in the German language |  |  |  |  |  |
| Very good | 1210 | 756 (62.50) | 1.75 (0.09)* | 68 (5.64)* | 0.51 (0.14) |
| Good | 1384 | 877 (63.39) | 1.86 (0.09) | 105 (7.58) | 0.43 (0.13) |
| Not bad | 1145 | 751 (65.57) | 2.07 (0.10) | 100 (8.75) | 0.89 (0.15) |
| Fairly bad | 411 | 273 (66.38) | 2.32 (0.16) | 40 (9.78) | 0.81 (0.25) |
| Not at all | 56 | 38 (67.65) | 1.71 (0.44) | 7 (13.08) | 0.94 (0.77) |

Comparisons of probabilities of at least one doctoral visit within three months and probabilities of at least one night in hospital within one year by sociodemographic characteristics were analyzed using F tests. Comparison of mean number of doctoral visits within three months and mean number of nights in hospital within one year by sociodemographic characteristics were analyzed using F tests.

SE: standard error

^a^ Without Russia, Türkiye, and Romania

^b^ Without East Europe

^c^ Without Kazakhstan

^d^ ‘Other main reason for migration’ is not shown

* p<0.05, ** p≤0.01, *** p≤0.001

Table S3: Model of self-reported visits to primary care physicians within three months (n = 4206; 15,837 observations), and self-reported number of nights in hospital within one year (n = 3711; 13,472 observations), and selected sociodemographic and migration-specific characteristics

| Variable | Model 3^†^ (dependent variable visit to primary care physicians within three months) | | | Model 4^‡^ (dependent variable number of nights in hospital within one year) | | |
| --- | --- | --- | --- | --- | --- | --- |
|  | OR | 95% CI | *p*-value | Exp(B) | 95% CI | *p*-value |
| Current health (Ref. very good) |  |  |  |  |  |  |
| Good | *1.57* | *1.39; 1.77* | *< 0.001* | 1.21 | 0.94; 1.55 | 0.145 |
| Satisfactory | *2.97* | *2.56; 3.43* | *< 0.001* | *2.28* | *1.72; 3.03* | *< 0.001* |
| Poor | *9.01* | *7.36; 11.03* | *< 0.001* | *4.72* | *3.34; 6.69* | *< 0.001* |
| Bad | *20.14* | *12.59; 32.21* | *< 0.001* | *7.79* | *5.28; 11.49* | *< 0.001* |
| Sex (Ref. male) |  |  |  |  |  |  |
| Female | 0.96 | 0.89; 1.04 | 0.337 | 0.91 | 0.78; 1.06 | 0.229 |
| Age, years | *1.02* | *1.01; 1.01* | *< 0.001* | 1.00 | 1.00; 1.01 | 0.285 |
| Marital status (Ref. married/in partnership) |  |  |  |  |  |  |
| Never married/single | *0.90* | *0.80; 1.00* | *0.043* | *0.67* | *0.54; 0.83* | *< 0.001* |
| Widowed | 1.01 | 0.84; 1.21 | 0.933 | *0.72* | *0.52; 0.99* | *0.040* |
| Separated/divorced | 0.97 | 0.82; 1.14 | 0.725 | 0.82 | 0.62; 1.10 | 0.191 |
| Employment (Ref. unemployed) |  |  |  |  |  |  |
| Employed | *0.62* | *0.56; 0.70* | *< 0.001* | *0.33* | *0.27; 0.39* | *< 0.001* |
| School-leaving qualification (Ref. secondary general school)^1^ |  |  |  |  |  |  |
| Secondary school | 0.99 | 0.87; 1.11 | 0.808 | 1.07 | 0.86; 1.33 | 0.563 |
| Academic secondary school | 0.99 | 0.88; 1.11 | 0.834 | 0.97 | 0.79; 1.19 | 0.789 |
| No school-leaving qualification | 0.87 | 0.65; 1.16 | 0.330 | 0.96 | 0.53; 1.75 | 0.896 |
| Religious affiliation (Ref. Christian) |  |  |  |  |  |  |
| Muslim | 0.94 | 0.78; 1.12 | 0.477 | 0.79 | 0.60; 1.05 | 0.102 |
| Other faith | 0.96 | 0.72; 1.29 | 0.795 | 0.73 | 0.47; 1.15 | 0.176 |
| Non-denominational | 0.93 | 0.81; 1.05 | 0.248 | 1.15 | 0.91; 1.45 | 0.239 |
| Time since migration to Germany, years | 1.00 | 0.99; 1.01 | 0.612 | 1.00 | 0.98; 1.01 | 0.767 |
| Country of birth (Ref. Russia) |  |  |  |  |  |  |
| Romania | 1.124 | 0.97; 1.58 | 0.080 | 1.09 | 0.76; 1.57 | 0.635 |
| Kazakhstan | 1.09 | 0.87; 1.36 | 0.475 | 1.08 | 0.74; 1.56 | 0.705 |
| Türkiye | *1.30* | *1.04; 1.63* | *0.022* | *1.51* | *1.05; 2.18* | *0.027* |
| Other East European country^2^ | *1.38* | *1.09; 1.75* | *0.008* | 1.09 | 0.74; 1.62 | 0.664 |
| Other European country^3^ | *1.41* | *1.15; 1.74* | *0.001* | 1.19 | 0.85; 1.67 | 0.314 |
| Other Asian country^4^ | *1.59* | *1.26; 2.01* | *< 0.001* | 1.35 | 0.90; 2.02 | 0.149 |
| African country | 1.29 | 0.93; 1.78 | 0.130 | 1.41 | 0.87; 2.30 | 0.161 |
| American/Oceanic country | 1.34 | 0.88; 2.04 | 0.168 | 0.75 | 0.37; 1.48 | 0.402 |
| German citizenship (Ref. yes) |  |  |  |  |  |  |
| No | 0.89 | 0.78; 1.02 | 0.084 | 1.09 | 0.87; 1.37 | 0.439 |
| Main reason for migration (Ref. family/partnership)^5^ |  |  |  |  |  |  |
| Economic reasons | 0.89 | 0.78; 1.01 | 0.074 | 1.15 | 0.94; 1.40 | 0.187 |
| Political reasons | 1.02 | 0.84; 1.24 | 0.846 | *0.74* | *0.56; 0.98* | *0.038* |
| Connectedness with country of birth (Ref. very strong) |  |  |  |  |  |  |
| Strong | *0.82* | *0.71; 0.94* | *0.006* | *1.29* | *1.01; 1.65* | *0.041* |
| In some respects | *0.85* | *0.73; 0.99* | *0.033* | *1.38* | *1.08; 1.76* | *0.011* |
| Hardly | *0.71* | *0.59; 0.85* | *< 0.001* | 1.09 | 0.80; 1.48 | 0.592 |
| Not at all | *0.64* | *0.52; 0.85* | *< 0.001* | 0.90 | 0.64; 1.27 | 0.561 |
| Feeling German (Ref. entirely) |  |  |  |  |  |  |
| Predominantly | 1.04 | 0.90; 1.21 | 0.594 | 0.98 | 0.75; 1.28 | 0.854 |
| In some respects | 1.06 | 0.91; 1.23 | 0.437 | 0.91 | 0.68; 1.22 | 0.547 |
| Hardly | 1.10 | 0.91; 1.33 | 0.318 | *0.74* | *0.52; 1.06* | *0.096* |
| Not at all | 1.02 | 0.82; 1.28 | 0.859 | 0.84 | 0.58; 1.22 | 0.355 |
| Disadvantages due to origin (Ref. often) |  |  |  |  |  |  |
| Rarely | 1.05 | 0.86; 1.28 | 0.624 | 0.87 | 0.60; 1.27 | 0.461 |
| Never | 0.98 | 0.80; 1.18 | 0.801 | 0.78 | 0.54; 1.12 | 0.172 |
| Oral ability in the German language (Ref. very good) |  |  |  |  |  |  |
| Good | 0.90 | 0.79; 1.02 | 0.095 | 1.14 | 0.90; 1.43 | 0.283 |
| Not bad | *0.79* | *0.68; 0.92* | *0.003* | 1.00 | 0.77; 1.30 | 0.987 |
| Fairly bad | *0.76* | *0.61; 0.94* | *0.013* | 1.02 | 0.71; 1.45 | 0.925 |
| Not at all | 0.62 | 0.35; 1.11 | 0.111 | 1.03 | 0.53; 2.02 | 0.922 |
| Survey year (Ref. 2014/2015^6^) |  |  |  |  |  |  |
| 2015 | 1.00 | 0.86; 1.15 | 0.977 | - | - | - |
| 2016 | 1.07 | 0.93; 1.23 | 0.333 | 1.00 | 0.78; 1.29 | 0.997 |
| 2017 | 1.13 | 0.98; 1.30 | 0.105 | 0.81 | 0.63; 1.03 | 0.085 |
| 2018 | 1.03 | 0.89; 1.20 | 0.680 | 0.82 | 0.60; 1.11 | 0.206 |
| 2019 | 1.08 | 0.92; 1.27 | 0.346 | *0.72* | *0.54; 0.95* | *0.021* |
| 2020 | *0.64* | *0.54; 0.76* | *< 0.001* | 0.76 | 0.56; 1.02 | 0.064 |
| Constant | 0.90 | 0.61; 1.33 | 0.605 | 0.79 | 0.40; 1.55 | 0.490 |

CI: confidence interval

^1^ ‘Other school-leaving qualification’ is not shown

^2^ Without Russia, Türkiye, and Romania

^3^ Without East Europe

^4^ Without Kazakhstan

^5^ ‘Other main reason for migration’ is not shown

^6^ As no data on the number of nights in hospital within one year was available for the year 2014, observations from the year 2014 were not used in model 4.

^†^ Random-effects panel-data logit model with cluster-robust standard errors

^‡^ Generalized linear panel-data model with gamma family and log-link function and cluster-robust standard errors for population-averaged panel data

Table S4: Model of zero-truncated self-reported number of visits to primary care physicians within three months (n = 3538; 10,248 observations), and zero-truncated self-reported number of nights in hospital within one year (n = 1174; 1764 observations), and selected sociodemographic and migration-specific characteristics

| Variable | Model 5^†^ (dependent variable number of zero-truncated visits to primary care physicians within three months) | | | Model 6^‡^ (dependent variable number of zero-truncated nights in hospital within one year) | | |
| --- | --- | --- | --- | --- | --- | --- |
|  | Exp(b) | 95% CI | *p*-value | Exp(b) | 95% CI | *p*-value |
| Current health (Ref. very good) |  |  |  |  |  |  |
| Good | *1.18* | *1.12; 1.25* | *< 0.001* | 0.98 | 0.79; 1.23 | 0.881 |
| Satisfactory | *1.56* | *1.46; 1.66* | *< 0.001* | 1.15 | 0.90; 1.46 | 0.275 |
| Poor | *2.27* | *2.10; 2.40* | *< 0.001* | *1.49* | *1.12; 1.99* | *0.006* |
| Bad | *3.74* | *3.23; 4.32* | *< 0.001* | *1.95* | *1.37; 2.78* | *< 0.001* |
| Sex (Ref. male) |  |  |  |  |  |  |
| Female | 1.01 | 0.98; 1.05 | 0.436 | 0.95 | 0.83; 1.09 | 0.449 |
| Age, years | *1.00* | *0.99; 1.00* | *0.003* | *1.01* | *1.00; 1.02* | *0.005* |
| Marital status (Ref. married/in partnership) |  |  |  |  |  |  |
| Never married/single | 1.01 | 0.96; 1.06 | 0.691 | *0.81* | *0.70; 0.95* | *0.007* |
| Widowed | 1.08 | 0.99; 1.18 | 0.088 | 0.91 | 0.73; 1.12 | 0.370 |
| Separated/divorced | 1.04 | 0.95; 1.13 | 0.370 | 0.94 | 0.75; 1.17 | 0.560 |
| Employment (Ref. unemployed) |  |  |  |  |  |  |
| Employed | *0.79* | *0.75; 0.82* | *< 0.001* | *0.75* | *0.64; 0.87* | *< 0.001* |
| School-leaving qualification (Ref. secondary general school)^1^ |  |  |  |  |  |  |
| Secondary school | 1.01 | 0.96; 1.06 | 0.721 | 1.01 | 0.85; 1.19 | 0.931 |
| Academic secondary school | 0.99 | 0.94; 1.04 | 0.616 | 1.09 | 0.94; 1.27 | 0.255 |
| No school-leaving qualification | 0.96 | 0.84; 1.10 | 0.554 | 1.55 | 0.92; 2.61 | 0.096 |
| Religious affiliation (Ref. Christian) |  |  |  |  |  |  |
| Muslim | 1.07 | 0.99; 1.17 | 0.101 | *0.75* | *0.62; 0.90* | *0.002* |
| Other faith | 1.01 | 0.89; 1.15 | 0.873 | 0.80 | 0.56; 1.13 | 0.202 |
| Non-denominational | 1.04 | 0.98; 1.10 | 0.205 | 1.12 | 0.94; 1.33 | 0.222 |
| Time since migration to Germany, years | 1.00 | 1.00; 1.00 | 0.853 | 1.00 | 0.99; 1.01 | 0.586 |
| Country of birth (Ref. Russia) |  |  |  |  |  |  |
| Romania | *1.20* | *1.09; 1.31* | *< 0.001* | 0.86 | 0.64; 1.16 | 0.330 |
| Kazakhstan | 1.08 | 0.97; 1.19 | 0.144 | 1.04 | 0.79; 1.36 | 0.783 |
| Türkiye | *1.19* | *1.09; 1.29* | *< 0.001* | 1.18 | 0.91; 1.53 | 0.215 |
| Other East European country^2^ | *1.18* | *1.08; 1.29* | *< 0.001* | 0.97 | 0.71; 1.31 | 0.834 |
| Other European country^3^ | *1.22* | *1.12; 1.32* | *< 0.001* | 1.02 | 0.79; 1.32 | 0.872 |
| Other Asian country^4^ | *1.16* | *1.06; 1.28* | *0.002* | 1.23 | 0.93; 1.62 | 0.146 |
| African country | 1.15 | 0.99; 1.33 | 0.061 | 1.00 | 0.74; 1.36 | 0.993 |
| American/Oceanic country | 1.11 | 0.97; 1.28 | 0.124 | 0.95 | 0.47; 1.91 | 0.890 |
| German citizenship (Ref. yes) |  |  |  |  |  |  |
| No | 1.02 | 0.97; 1.07 | 0.509 | 1.03 | 0.88; 1.20 | 0.717 |
| Main reason for migration (Ref. family/partnership)^5^ |  |  |  |  |  |  |
| Economic reasons | 1.00 | 0.94; 1.05 | 0.902 | 1.03 | 0.87; 1.21 | 0.760 |
| Political reasons | 0.97 | 0.88; 1.06 | 0.495 | 0.84 | 0.68; 1.03 | 0.098 |
| Connectedness with country of birth (Ref. very strong) |  |  |  |  |  |  |
| Strong | *0.92* | *0.87; 0.98* | *0.010* | 1.13 | 0.96; 1.34 | 0.141 |
| In some respects | 0.95 | 0.89; 1.02 | 0.159 | *1.34* | *1.10; 1.62* | *0.003* |
| Hardly | *0.92* | *0.85; 0.99* | *0.035* | 1.17 | 0.93; 1.48 | 0.167 |
| Not at all | 0.98 | 0.87; 1.10 | 0.746 | 1.02 | 0.79; 1.31 | 0.907 |
| Feeling German (Ref. entirely) |  |  |  |  |  |  |
| Predominantly | 0.97 | 0.90; 1.04 | 0.330 | 1.12 | 0.94; 1.33 | 0.202 |
| In some respects | 1.00 | 0.93; 1.07 | 0.958 | 1.05 | 0.88; 1.26 | 0.562 |
| Hardly | 0.99 | 0.91; 1.09 | 0.903 | 0.99 | 0.77; 1.28 | 0.958 |
| Not at all | 1.02 | 0.92; 1.12 | 0.751 | 0.93 | 0.74; 1.18 | 0.547 |
| Disadvantages due to origin (Ref. often) |  |  |  |  |  |  |
| Rarely | 1.04 | 0.96; 1.13 | 0.333 | 0.93 | 0.68; 1.27 | 0.651 |
| Never | 1.01 | 0.93; 1.09 | 0.864 | 0.81 | 0.60; 1.09 | 0.164 |
| Oral ability in the German language (Ref. very good) |  |  |  |  |  |  |
| Good | 0.97 | 0.92; 1.02 | 0.196 | 1.02 | 0.86; 1.22 | 0.803 |
| Not bad | 0.95 | 0.90; 1.01 | 0.110 | 1.01 | 0.84; 1.22 | 0.908 |
| Fairly bad | 0.95 | 0.86; 1.04 | 0.272 | 1.00 | 0.79; 1.28 | 0.976 |
| Not at all | 1.05 | 0.66; 1.67 | 0.836 | 0.99 | 0.67; 1.47 | 0.958 |
| Survey year (Ref. 2014/2015^6^) |  |  |  |  |  |  |
| 2015 | 1.02 | 0.96; 1.09 | 0.522 | - | - | - |
| 2016 | 1.00 | 0.93; 1.06 | 0.879 | 0.96 | 0.79; 1.18 | 0.714 |
| 2017 | 0.95 | 0.90; 1.02 | 0.145 | 0.86 | 0.73; 1.02 | 0.080 |
| 2018 | 0.98 | 0.92; 1.04 | 0.518 | 0.96 | 0.76; 1.21 | 0.719 |
| 2019 | 0.99 | 0.93; 1.05 | 0.721 | *0.78* | *0.64; 0.95* | *0.013* |
| 2020 | 0.94 | 0.87; 1.01 | 0.085 | 0.88 | 0.70; 1.11 | 0.281 |
| Constant | *2.23* | *1.90; 2.62* | *< 0.001* | *4.85* | *2.81; 8.36* | *< 0.001* |

CI: confidence interval

^1^ ‘Other school-leaving qualification’ is not shown

^2^ Without Russia, Türkiye, and Romania

^3^ Without East Europe

^4^ Without Kazakhstan

^5^ ‘Other main reason for migration’ is not shown

^6^ As no data on the number of nights in hospital within one year was available for the year 2014, observations from the year 2014 were not used in model 6.

^†^ Random-effects panel-data logit model with cluster-robust standard errors

^‡^ Generalized linear panel-data model with gamma family and log-link function and cluster-robust standard errors for population-averaged panel data
